# Anti-HSV-1 activity of *Aspergillipeptide* D, a cyclic pentapepetide isolated from fungus *Aspergillus* sp. SCSIO 41501

**DOI:** 10.1186/s12985-020-01315-z

**Published:** 2020-03-19

**Authors:** Zhaoyang Wang, Jiaoyan Jia, Lu Wang, Feng Li, Yiliang Wang, Yuzhou Jiang, Xiaowei Song, Shurong Qin, Kai Zheng, Ju Ye, Zhe Ren, Yifei Wang, Shuhua Qi

**Affiliations:** 1 Guangzhou Jinan Biomedicine Research and Development Center, National Engineering Research Center of Genetic Medicine, Jinan University, Guangzhou, Guangdong China; 2 School of Pharmaceutical Sciences, Health Science Center, Shenzhen University, Shenzhen, China; 3 Key Laboratory of Plant Chemistry in Qinghai-Tibet Plateau, Qinghai University for Nationalities, Xining, 810007 Qinghai China; 4 CAS Key Laboratory of Tropical Marine Bio-resources and Ecology, South China Sea Institute of Oceanology Chinese Academy of Sciences, 164 West Xingang Road, Guangzhou, 510301 Guangdong China

**Keywords:** HSV-1, *Aspergillipeptide* D, Marine peptide, Glycoprotein B

## Abstract

**Background:**

Herpes simplex virus 1, an enveloped DNA virus belonging to the Herpesviridae family, spreads to neurons and causes pathological changes in the central nervous system. The purpose of this study was to investigate the potency and mechanism of antiviral activity of *Aspergillipeptide* D, a cyclic pentapeptide isolated from a culture broth of marine gorgonian-derived fungus *Aspergillus sp. SCSIO 41501*, At present, there are many studies on the anti-tumor, anti-clotting, anti-oxidant and immunoinflammatory effects of *Aspergillipeptide* D, but little research has been done on the anti-HSV-1 activity of *Aspergillipeptide* D.

**Methods:**

The anti-HSV-1 activity of *Aspergillipeptide* D was evaluated by plaque reduction assay. The mechanism of action against HSV-1 was determined from the effective stage. Then we assayed the viral DNA replication, viral RNA synthesis and protein expression, respectively. We also identified the proteins that interact with gB by mass spectrometry, and assayed the effect of *Aspergillipeptide* D on the interaction between the virus gB protein and cell proteins.

**Results:**

Plaque reduction experiments showed that *Aspergillipeptide* D did not affect HSV-1 early infection events, including viral inactivation, attachment and penetration. Interestingly, *Aspergillipeptide* D dramatically reduced both the gene and protein levels of viral late protein gB, and suppressed its location in the endoplasmic reticulum and Golgi apparatus. In contrast, overexpression of gB restored viral production. Finally, proteomic analysis revealed that the numbers of cellular proteins that interacted with gB protein was largely decreased by *Aspergillipeptide* D. These results suggested that *Aspergillipeptide* D inhibited gB function to affect HSV-1 intercellular spread.

**Conclusions:**

Our results indicated that *Aspergillipeptide* D might be a potential candidate for HSV-1 therapy, especially for ACV-resistant strains.

## Background

Herpes simplex virus 1 (HSV-1), an enveloped DNA virus belonging to the Herpesviridae family, spreads to neurons and causes pathological changes in the central nervous system [1]. HSV-1 virus particle consists of a core and a linear double-stranded DNA enclosed in a capsid; an outer envelope containing various glycoproteins covers tegument proteins, which are exterior to the viral capsid [2]. HSV-1 envelopes contain at least 14 different proteins [3], but only four of them, gB, gD, gH and gL are required for entry, which are established by analyzing the infectivity of HSV-1 mutants containing single gene deletions [4–7] . gB is the most highly conserved glycoprotein, and is a class III viral fusion protein involved directly in the viral and host cell membrane interaction and fusion [8, 9].

Nowadays most of antiviral drugs applied in clinic are largely nucleic acid analogs, all of which target viral DNA replication process. One representative example is Acyclovir (ACV). As a consequence, drug-resistant HSV strains, especially ACV-resistant HSV strains, found frequently [10]. Therefore, the development of novel anti-HSV agents with different mechanisms of action is urgent.

**Fig. 1:**
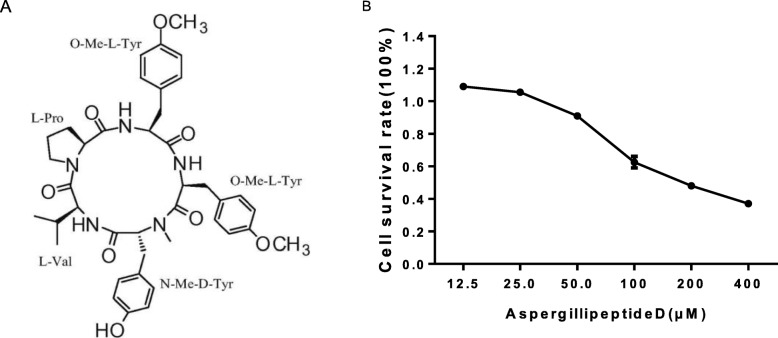
Cytotoxicity of *Aspergillipeptide* D. **a** The structure formula of *Aspergillipeptide* D. **b** Vero cells were treated with different concentrations of *Aspergillipeptide*D for 48 h, and MTT method was performed to calculate the cell survival rate. Data are mean ± SD (*n* = 3)

Many marine peptides, obtained from seaweeds, fishes, mollusk, crustaceans, crabs and marine bacteria and fungi, show various biological activities such as anti-tumor, anti-virus, anti-oxidant, immunoinflammatory effects and other pharmaceutical properties based on their structural characteristics, amino acid composition and sequences [11–13]. In our previous study, *Aspergillipeptide* D, a new cyclic pentapeptide, was obtained from the fungal strain *Aspergillus* sp. SCSIO 41501 as white solid with the molecular formula of C40H49N5O8 (Fig. 1a) [14]. In this study, we further investigated the potency and mechanism of antiviral activity of *Aspergillipeptide* D against HSV-1 and ACV-resistant strains.

## Main text

### Methods

#### Chemicals and reagents

The cyclic pentapeptide, *Aspergillipeptide* D, was isolated from the fungal strain Aspergillus SCSIO 41501 [14]. ACV (acyclovir) and 2-(2,5-dimethyl-2-thiazolyl)-2,5-diphenyl-2H-tetrazolium bromide (MTT) were obtained from SigmaAldrich (St. Louis, MO, USA). Trizol Reagent was purchased from Invitrogen (Carlsbad, CA, USA). Dulbecco’s modified Eagle medium (DMEM), fetal bovine serum (FBS), and penicillin-streptomycin were bought from Gibco-BRL (Gland Island, NY, USA). *Aspergillipeptide* D and ACV were dissolved in dimethylsulfoxide (DMSO), and the final concentrations of DMSO were less than 0.1%. Restriction enzymes were purchased from Takara Bio (Shiga, Japan).

#### Cells and viruses

African green monkey kidney cells (Vero; ATCC CCL81) were cultured in DMEM supplemented with 10% heat-inactivated FBS. The maintenance medium used for virus dilutions was DMEM supplemented with 2% heat-inactivated FBS. HSV-1/F (ATCC VR-733) was preserved in our lab. HSV-1/Blue, a TK mutant derived from HSV-1 (KOS) [15], two ACV-resistant clinical HSV-1 strains (HSV-1/106 and HSV-1/153) were a kind gift from Tao Peng (Guangzhou Institutes of Biomedicine and Health, Chinese Academy of Sciences). All viruses were propagated in Vero cells and stored at − 80 °C until further use.

#### MTT assay

The MTT assay was performed according to the standard protocol. Briefly, Vero cells were cultured in 96-well plates. After the cell confluence reached 90%, various concentrations of compound were added to the plate, with each concentration having three replicates. After 48 h of incubation, 10 μl MTT solution (5 mg/mL) was added to each well, and the plate was incubated for 4 h in the dark. Then, the MTT solution was discarded, and 100 μl DMSO was added to each well. Plates were incubated for 15 min at room temperature with gently shaking. The optical density (OD) at 570 and 630 nm was measured for each well with an enzyme immunoassay reader (Bio-Rad, Hercules, CA, USA). The 50% cytotoxicity concentration (CC50) was defined as the concentration to reduce 50% cell viability.

#### Viral titer determination using plaque assay

Vero cells were cultured in 96-well plates. And the next day ten-fold serial dilutions of with and without treatment of extracts of HSV-1 were prepared prior to infection. Vero cell monolayers were then infected with different dilutions of 100 μl HSV-1 and allowed to adsorb for 2 h at 37 °C and 5% CO_2_. Unabsorbed viruses were aspirated, and plates were then overlaid with a nutrient medium-containing agar and incubated at 37 °C and 5% CO_2_ for 3 days. Plaques were visualized by staining cells with crystal violet and counting within 50 h. The plaque assay was carried out in triplicate. Virus was quantified by serial dilution and titration assay. The TCID50 (50% tissue culture infectious dose) was calculated using the formula of Reed and Muench method [16]:

Log10 50% end point dilution = log10 of dilution showing a mortality next above 50% - (difference of logarithms × logarithm of dilution factor).

Difference of logarithms = [(mortality at dilution next above 50%)-50%] / [(mortality next above 50%) - (mortality next below 50%)].

#### Plaque reduction assay

Experimental wells of 24-well plates containing confluent monolayers of Vero cells were infected with virus suspensions to produce 50 plaques per well. After 2 h incubation at 37 °C and 5% CO2, unabsorbed virions were aspirated. *Aspergillipeptide* D solution (25 μM, 12.5 μM, 6.25 μM, 3.125 μM, 1.5625 μM, and 0.78125 μM, respectively) was then added to the appropriate wells, followed by nutrient medium containing agar; the plates were incubated at 37 °C and 5% CO2 for 3 days. Plaques were counted as described above. The antiviral activity was calculated by the following formula:

$$ \mathrm{Antiviral}\ \mathrm{activity}\ \left(\%\right)=\frac{\mathrm{plaque}\ \mathrm{number}\left(\mathrm{control}\right)-\mathrm{plaque}\ \mathrm{number}\left(\mathrm{assay}\right)}{\mathrm{plaque}\ \mathrm{number}\left(\mathrm{control}\right)}\times 100\% $$

#### Virus inactivation assay

Culture Vero cells into 24-well plates (1.5*10^5^ cells/well), and the next day 100 μl of virus inoculum (50 PFUs per well) and 100 μl of *Aspergillipeptide* D solution (different concentrations) were mixed and incubated for 2 h at 37 °C. Then the mixture was added into cell wells and incubated at 37 °C for 2 h. The inoculated were removed. Cells were replenished with cover layer and 3 days later were fixed, stained as described above.

#### Virus attachment assay

Culture Vero cells into 24-well plates (1.5*10^5^ cells/well), and the next day cells were pre-cooled at 4 °C for 1 h and washed by cold PBS. Virus inoculum (50 PFUs per well) and *Aspergillipeptide* D at indicated concentrations were added into cell wells, and the mixture was incubated at 4 °C for another 2 h to allow virus attaching to the cells. The virus inoculum was removed. Cells were replenished with cover layer and 3 days later were fixed, stained as described above.

#### Virus penetration assay

Culture Vero cells into 24-well plates (1.5*10^5^ cells/well), and the next day cells were pre-cool at 4 °C for 1 h and washed by cold PBS then infected by virus (50 PFUs per well) for another 2 h at 4 °C to allow virus attaching to the cells. After that, the virus inoculum was removed, and cells were washed by cold PBS. Then different concentrations of *Aspergillipeptide* D were added and incubated at 37 °C for 10 min to maximize virus penetration. After incubation, PBS (pH = 3) was added into every well for 1 min to inactive the virus which failed to penetrate the cells. After that, the solution was neutralized and the neutral PBS was removed. Cells were replenished with cover layer and 3 days later were fixed, stained as described above.

#### Treatment effects after virus infection

Vero cells were cultured in 24-well plates. And next day cells were infected with HSV-1 (50 PFUs per well) for 2 h at 37 °C. After infection, the virus inoculum was removed, and cells were washed by PBS, and overlaid with *Aspergillipeptide* D at the indicated concentrations. After 3 days, cells were fixed, stained as described above.

#### The analysis of HSV-1 DNA synthesis

Vero cells were cultured in 24-well plates. And next day cells infected with HSV-1 (MOI = 3) were incubated with or without *Aspergillipeptide* D (25 μM) for 15 h. Viral DNA was extracted using GeneJET Viral DNA and RNA Purification Kit (Thermo). RT-PCR assay was used to quantify the viral DNA. Then the HSV-1 genome copy numbers were expressed relative to the virus control groups. The primer pairs are as follow: *UL47* (F: 5′-GACGTA CGCGAT GAG ATC AA -3′, R: 5′-GTT ACC GGA TTA CGG GGA CT-3′).

#### Real-time PCR

Vero cells were cultured in 6-well plates. And next day cells infected with HSV-1 (MOI = 3) were incubated with *Aspergillipeptide* D (25 μM) for 3, 6, 9 h, respectively. Total RNA was isolated using Trizol (Invitrogen) and subjected to cDNA synthesis using a PrimeScript RT reagent kit (Takara). Real-time PCR (RT-PCR) was conducted to determine the expression levels of immediate early (IE) gene *UL54*, early (E) gene *UL52* and late (L) gene *UL27* of HSV-1/F and HSV-1/106 at 3, 6 and 9 h pi., respectively. The primer pairs were the same as described before [17].

#### Immunofluorescence assay

Vero cells were cultured in confocal dish, next day cells infected with HSV-1 (MOI = 3) at 37 °C for 2 h for viral adsorption. Cells were transferred into main medium with or without 25 μM *Aspergillipeptide*s D and incubated for 9 h.p.i. Cells were fixed for 15 min with 4% paraformaldehyde (PFA) and permeabilized with 0.02% Triton X-100, both in PBS, and subsequently incubated with anti-gB antibody (Abcam) for 60 min and Alexa Fluor 488(1:1000) secondary antibody (Invitrogen) for 60 min. Then, the cells were stained with Golgi-Tracker Red or ER-Tracker Red (Beyotime, China). After each step the slides were washed repeatedly with PBS, and finally they were preserved with PBS. The additional nuclear staining with 4,6-diamidino-2-phenylindole (DAPI, Molecular Probes) was per-formed for 20 min. Fluorescence was recorded in a confocal laser scan microscope (LSM 510 meta; Zeiss) [17].

#### Western blotting

Vero cells were seeded in 60 mm cell culture dish with the density of 1.5 × 10^6^ cells/ dish. After 24 h, cells were infected with HSV-1 (MOI = 3) at 37 °C for 2 h. DMEM maintenance medium containing *Aspergillipeptide*s D (25 μM) was added. At 6 and 9 h post-infection, the cells were washed three times with PBS, and were lysed using RIPA buffer (Beyotime). The equal amount (40 μg/sample) proteins were subjected to Western Blot analysis. A primary antibody against HSV-1 ICP0 (abcam1:1000), ICP8 (abcam 1:8000), VP5 (santa1:1000), gB (abcam1:1000) and gD (abcam1:1000) was used to detect the content changes of immediate early, early and late protein [18].

#### Co-immunoprecipitation (co-IP) and LC-MS analysis

Vero cells were seeded in 100 mm cell culture dish with the density of 3 × 10^6^ cells/ dish. After 24 h, cells were treated with *Aspergillipeptide* D (25 μM) and infected with HSV-1 (MOI = 3) for 9 h. The cells were then lysed and the protein concentrations were measured and adjusted to 1 mg/ml. The lysate was precleared by adding 1.0 μg of the appropriate control IgG (normal mouse or rabbit IgG, corresponding to the host species of the primary antibody), together with 20 μl of resuspended volume of Protein A/G PLUSA agarose. Afterwards, the mixture was incubated at 4 °C for 30 min. The optimal dilution of primary antibody was added to the cell lysates (supernatant), incubated for 1 h at 4 °C, and then incubated at 4 °C overnight with 30 μl of resuspended volume of Protein A/G PLUS-Agarose. Next, the immunoprecipitation was collected, washed with PBS, and resuspended in 20 μl 1 × SDS PAGE buffer (Beyotime, China). The LC-MS Analysis were provided by the BGI (China).

#### Statistical analysis

Results were calculated as the mean ± SD, and statistical significance were determined by the Student’s t test. *P* values (P)<0.05 were considered statistically significant.

## Results

### Cytotoxicity and anti-HSV-1 activity of *Aspergillipeptide* D

**Fig. 2:**
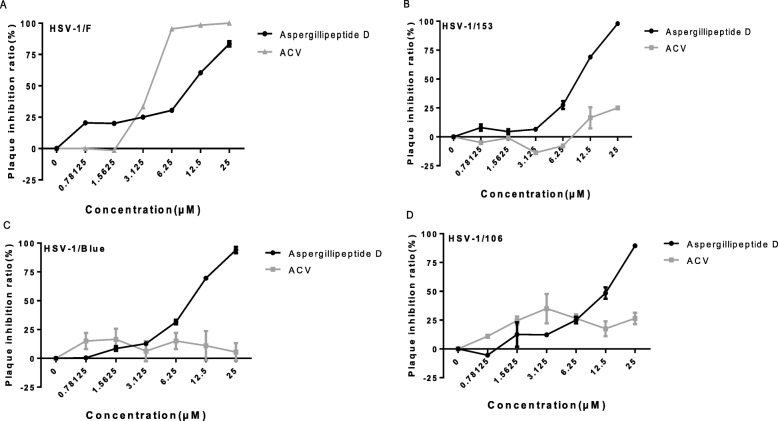
Antiviral activities of *Aspergillipeptide* D against HSV-1 and ACV-resistant strains. Plaque reduction assay was used to evaluate the antiviral activity of *Aspergillipeptide* D against HSV-1/F, a standard experimental strains (**a**); HSV-1/blue, a TK mutant derived from HSV-1 (KOS) (**c**); HSV-1/106 and HSV-1/153, two ACV-resistant clinical HSV-1 strains, respectively (**b**, **d**). Vero cells were infected with HSV-1, HSV-1/153, HSV-1/Blue and HSV-1/106 (MOI = 0.1) at 37 °C for 2 h. The supernatant was then discarded, and the cover fluid containing with *Aspergillipeptide* D was added. After 72 h, the cells were fixed, dyed, and the plaque inhibition rate was calculated. Data are mean ± SD (*n* = 3)

To examine the cytotoxic effect of *Aspergillipeptide* D on Vero cells, MTT assay was used. A significant reduction of cell vitality was observed at concentration > 25 μM, corresponding with the CC50 value as 208.723 ± 9.717 μM (Fig. 1b). Next, the antiviral activities of *Aspergillipeptide* D against HSV-1/F and three ACV-resistant strains, including HSV-1/Blue, a TK mutant derived from HSV-1, and two clinical HSV-1 strains HSV-1/106 and HSV-1/153 [19], were monitored by plaque reduction assay (Fig. 2), which clearly showed that *Aspergillipeptide* D inhibits both HSV-1/F and ACV-resistant strains infection in a dose-dependent manner. As shown in Table 1, the 50% effective concentrations (EC50) for *Aspergillipeptide* D and ACV to inhibit HSV-1/F were 7.928 ± 0.511 μM and 3.606 ± 0.302 μM, respectively. The EC50 values of *Aspergillipeptide* D against HSV-1/153, HSV-1/106, and HSV-1/Blue were 8.277 ± 1.249 μM, 10.486 ± 0.929 μM, and 7.9875 ± 0.616 μM, respectively. In contrast, the EC50 of ACV against all the three resistant strains were more than 40 μM. Together, these results indicated that *Aspergillipeptide* D has a significant antiviral effect against ACV-resistant HSV-1 strains.

**Table 1:** Antiviral activity of ACV and *Aspergillipeptide* D

Compound	CC50^b^(μM)	EC50^c^(μM)			
		HSV-1/F	HSV-1/106	HSV-1/153	HSV-1/Blue
ACV^a^	> 500	3.606 ± 0.302	46.234 ± 15.335	85.746 ± 21.753	273.742 ± 20.826
*Aspergillipeptide* D	208.723 ± 9.717**	7.928 ± 0.511	10.486 ± 0.929**	8.277 ± 1.249**	7.9875 ± 0.616**

^a^ Acyclovir

^b^ The CC50 (50% cytotoxic concentration for Vero cells in lg/ml); mean ± S.E

^c^ The EC50 (Concentration of compound producing 50% inhibition of virus-induced cytopathic effect)

**represents a significant difference compered with the control group

### Mode of antiviral activity

**Fig. 3:**
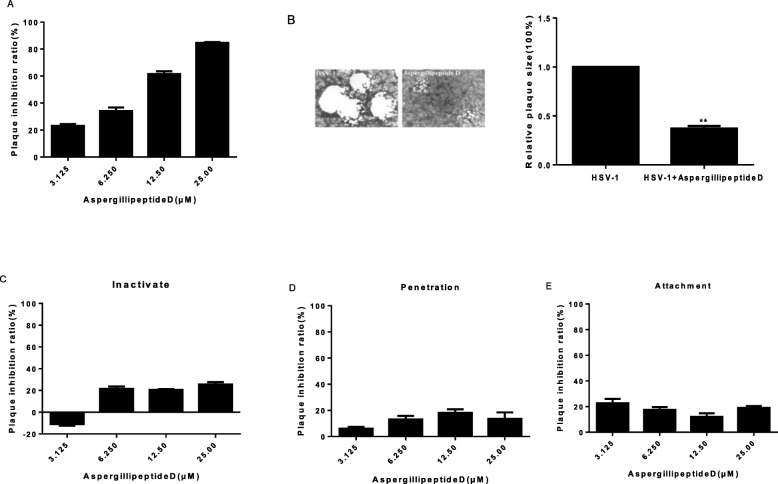
*Aspergillipeptide* D affects HSV-1 late infection. **a**-**b***Aspergillipeptide* D reduces HSV-1 production and plaque size. The cells seeded in 24-well plates were treated with HSV-1 and *Aspergillipeptide* D for 2 h. The supernatant was then removed, and the cover fluid containing with *Aspergillipeptide* D was added. After 72 h, the cells were fixed, dyed, and the plaque inhibition rate was calculated. The size of plaque was measured and analyzed. Data are mean ± SD (*n* = 3), **p<0.01. **c**-**e***Aspergillipeptide* D has no significant effect on viral inactivation (**c**), penetration (**d**) and attachment (**e**), Data are mean ± SD (*n* = 3) [20]

Next, we analyzed which step of HSV-1 life cycle was affected by *Aspergillipeptide* D. Plaque assay was performed to demonstrate that *Aspergillipeptide* D significant reduced HSV-1 infection in a dose-dependent manner (Fig. 3a). Besides, the HSV-1-induced plaque size was largely reduced (Fig. 3b), implying the decreased virion production. Then viral inactivation assay, viral penetration assay, and viral attachment assay were performed, which showed that *Aspergillipeptide* D had no significant effect on viral inactivation, attachment and penetration (Fig. 3c-e). These results suggested that *Aspergillipeptide* D affected viral late infection events, such as replication and release.

### Effects of *Aspergillipeptide* D on viral gene and protein expression

**Fig. 4:**
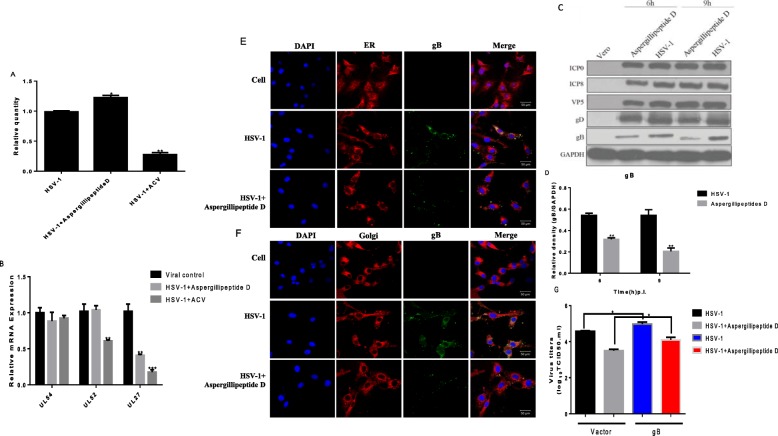
*Aspergillipeptide* D affects the expression and localization of viral Glycoprotein B. **a** the effect of *Aspergillipeptide* D on viral DNA synthesis. Vero cells infected with HSV-1 (MOI = 3) were incubated with or without *Aspergillipeptide* D (25 μM) for 15 h. The total DNA was extracted, and the expression level was detected. **b** the mRNA expression levels of viral immediate early gene *UL54*, early gene *UL52* and late gene *UL27* at 3, 6 and 9 h post-infection, respectively. Data are mean ± SD (*n* = 3). **p < 0.01 ***p < 0.001 versus HSV-1-treated group. **c***Aspergillipeptide* D reduced the protein level of HSV-1 gB. Vero cells were infected with HSV-1 for the indicated times and cell lysates were subjected to western blot assay for different HSV-1 proteins. **d** Densitometric analysis for gB western blot bands was shown. GAPDH served as a loading control. Data are mean ± SD (*n* = 3). **p < 0.01 versus HSV-1-treated group. **e-f** Immunofluorescence experiments demonstrated the location of gB in endoplasmic reticulum (**e**) and the Golgi apparatus (**f**) under the treatment of *Aspergillipeptide* D. Vero cells seeded in confocal dishes were treated with HSV-1(MOI = 3) or *Aspergillipeptide* D for 9 h. The cells were then stained with ER-Tracker Red (Red), Golgi-Tracker Red (red), DAPI (nucleus, blue) and anti-gB primary antibody (green). **g** Vero cells transfected with HA-labeled gB plasmid were infected with HSV-1 for 48 h in the presence or absence of *Aspergillipeptide* D. The virus titer was then measured

To determine whether *Aspergillipeptide* D affected viral DNA replication, viral DNA was extracted from Vero cells treated with or without *Aspergillipeptide* D, and the copy number of UL47 was examined. As shown in (Fig. 4a), *Aspergillipeptide* D didn’t affect the production of UL47. To analyze the effects of *Aspergillipeptide* D on HSV-1 gene expression, the mRNA expression levels of viral immediate-early gene (*UL54*), early gene (*UL52*), and late gene (*UL27*) were quantified at 3, 6, and 9 h pi., respectively (Fig. 4b). Interestingly, *Aspergillipeptide* D significantly reduced the expression of *UL27*, without significant effect on the expression of *UL54* and *UL52*, Consistently, western blotting assay demonstrated that *Aspergillipeptide* D reduced the protein level of viral late protein gB (encoded by viral late gene *UL27*) and did not affect viral immediately early protein ICP0, early protein ICP8, late protein VP5 and gD (Fig. 4c). Furthermore, immunofluorescent assay showed that the expression and the localization of gB in the endoplasmic reticulum and Golgi apparatus was largely reduced with the treatment of *Aspergillipeptide* D (Fig. 4e and f). Considering the fact that *Aspergillipeptide* D had no significant effect on viral attachment and penetration (Fig. 3c-f), it is reasonable to infer that *Aspergillipeptide* D inhibited gB to influence viral assembly, release and intercellular spread, as illustrated by the viral reduced plaque size (Fig. 3b). To confirm whether *Aspergillipeptide* D exerted its antiviral activity through gB, we constructed a HA-tagged gB plasmid and tested the effect of overexpression of gB on viral titer (Fig. 4g). Indeed, gB overexpression obviously enhanced HSV-1 infection and restored some of the virus production reduced by *Aspergillipeptide* D. In summary, these above results suggested that *Aspergillipeptide* D reduces the expression and location of viral gB to affect HSV-1 infection.

### Effect of *Aspergillipeptide* D on the interaction between the viral gB and cellular proteins

**Fig. 5:**
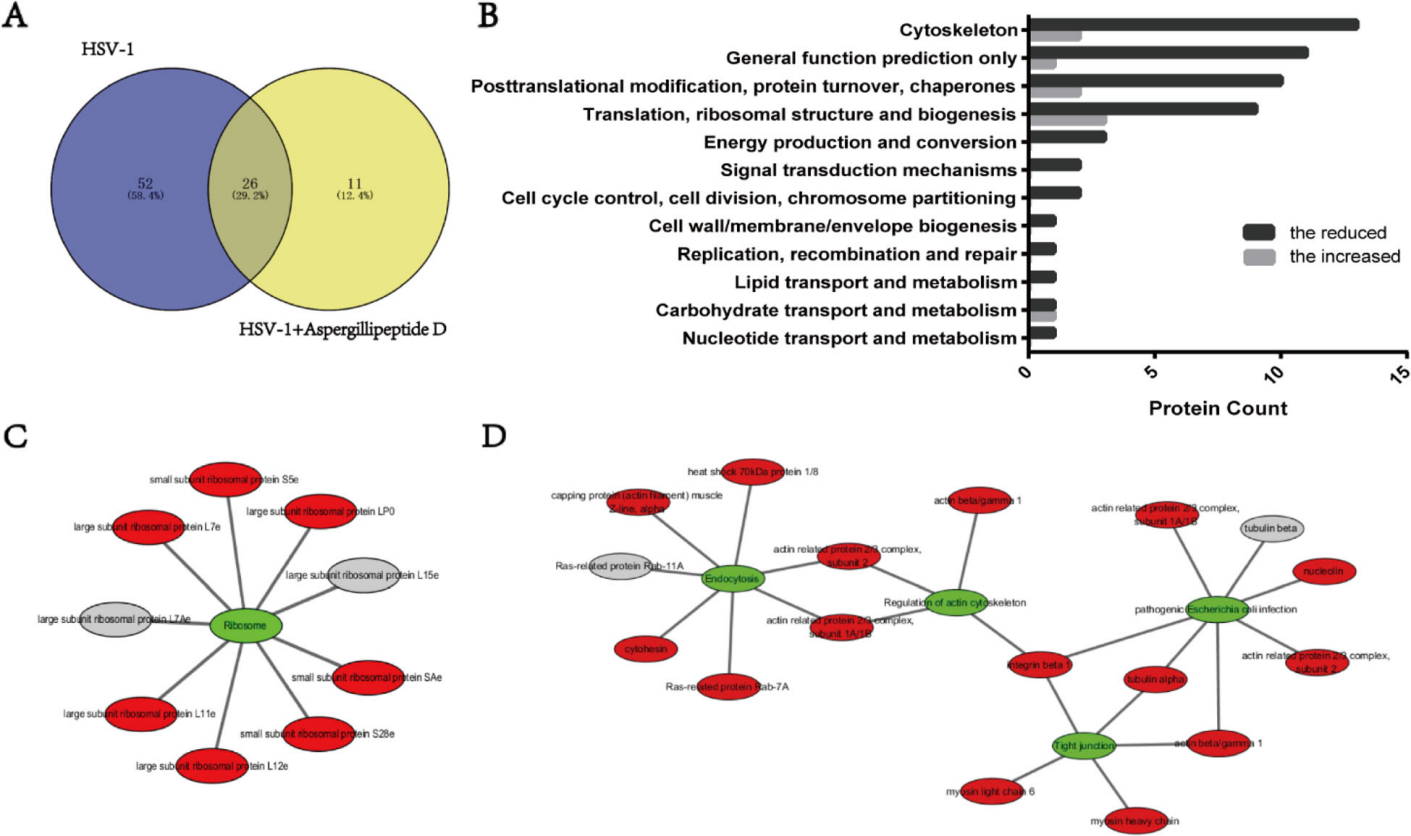
*Aspergillipeptide* D reduces cellular proteins interacted with viral gB. **a** Venn diagram showed the number of proteins interacted with gB with or without *Aspergillipeptide* D treatment. **b** COG classifications of the reduced or increased proteins interacted with gB in *Aspergillipeptide* D treatment. c-d KEGG Pathway analysis of the reduced or increased proteins, visualized by Cytoscape. Red represents the reduced proteins, and gray represents the increased proteins

Finally, we performed proteomic analysis to evaluate the effect of *Aspergillipeptide* D on gB. Different proteins interacted with gB during viral late stage were extracted by co-immunoprecipitation (Co-IP), and were then identified by mass spectrometry (MS). According to the Venn diagram, there were 78 proteins interacted with gB protein in HSV-1 infection, the number of which was reduced to 37 in the presence of *Aspergillipeptide* D (Fig. 5a). Next, the COG (Cluster of Orthologous Groups of Proteins) annotation analysis was performed to predict the possible functions of these proteins, and we found that the reduction of gB-interacted proteins by *Aspergillipeptide* D were mainly involved in translation, ribosomal structure, biogenesis, posttranslational modification, protein turnover, chaperones and cytoskeleton (Fig. 5b). In addition, KEGG pathways enrichment analysis indicated that these reduced gB-interacted proteins were mainly enriched in pathways associated with ribosome (Fig. 5c), tight junction, regulation of actin cytoskeleton and endocytosis (Fig. 5d). Among these proteins, integrin beta 1 that plays critical roles in three pathways maybe a key protein.

## Discussion

Oceans provide tremendous resources for the discovery of potential therapeutic agents. In the last few decades, many interesting compounds have been found in marine organisms [21, 22]. Herein, we demonstrated that *Aspergillipeptide* D, a new cyclic pentapeptide obtained from the fungal strain Aspergillus sp. SCSIO 41501 [23], exhibited obvious antiviral activity against HSV-1 and ACV-resistant strains. We also investigated its possible mechanisms of antiviral action.

Plaque reduction experiments showed that *Aspergillipeptide* D had a significant antiviral effect in a concentration-dependent manner (Fig. 3a), but without significant effect HSV-1 early infection events, including inactivation, attachment and penetration. More detailed studies indicated that *Aspergillipeptide* D significantly reduced the gene and protein levels of viral late protein gB (Fig. 4). In addition, *Aspergillipeptide* D reduced the localization of gB in the endoplasmic reticulum and the Golgi apparatus.

Entry into target cells is the first step for virus infection [24]. The core entry machinery for herpesviruses is formed by the gH, gD, gL and gB proteins. The herpesvirus gB protein is a class III viral fusion protein [25, 26]. Once gB is triggered, its fusion loops (FLs) insert into the target host cell lipid bilayer, followed by gB refolding to drive membrane merger and the onset of infection [26]. Our results showed that *Aspergillipeptide* D had no significant effect on viral inactivation, attachment and penetration, but could significantly reduce the expression of gB protein. These results suggested that *Aspergillipeptide* D affected the synthesis of gB protein to reduce HSV-1 intercellular spread, as viral plaque size was largely reduced (Fig. 3b). Such antiviral mechanism of *Aspergillipeptide* D is different from that of ACV, which may be responsible for the significant antiviral effects of *Aspergillipeptide* D on ACV-resistant strains (HSV-1/106, HSV-1/ Blue and HSV-1/153).

To further analyze the effect of *Aspergillipeptide* D on gB, cellular proteins that interact with gB protein were identified (Fig. 5). *Aspergillipeptide* D significantly reduced the gB-interacted proteins involved in translation, ribosomal structure, biogenesis; posttranslational modification, protein turnover, chaperones and cytoskeleton. In addition, KEGG pathways enrichment analysis indicated that these proteins were mainly enriched in pathways associated with ribosome, tight junction, regulation of actin cytoskeleton, pathogenic *E. coli* infection and endocytosis. Among them, integrin beta 1 that plays critical roles in three pathways maybe a key protein. Integrins are cell surface heterodimeric glycoproteins that contribute to a variety of functions, including cell-cell and cell-matrix adhesion and induction of signal transduction pathways [27]. Recently, it has been demonstrated that integrin beta 1 accumulated and formed a complex with CD98hc at the nuclear membrane in HSV-1-infected cells (26). Knockdown of integrin beta 1 induced aberrant accumulation of primary enveloped virions in the perinuclear space and in the membranous invagination structures adjacent to the nuclear membrane, implying that integrin beta 1 playing critical role in HSV-1 nuclear egress and assembly [28]. Further works are inspired to investigate whether *Aspergillipeptide* D affects the possible function of integrin beta 1 in HSV-1 late infection.

## Conclusion

Summarizing these data, *Aspergillipeptide* D is assessed to be a potential candidate for HSV-1 therapy, especially for ACV-resistant strains.

## Data Availability

All data from the current study are available from the corresponding author on request.

## Abbreviations

ACV: Acyclovir
Co-IP: Coimmunoprecipitation
CPE: Cytopathic effect
DMEM: Dulbecco’s modified Eagle’s medium
DMSO: Dimethyl sulfoxide
HSV-1: Herpes simplex virus 1
MOI: Multiplicity of infection
PBS: Phosphate-buffered saline
TCID_50_: 50% tissue culture infective dose
