# Correction to: Anti-HSV-1 activity of Aspergillipeptide D, a cyclic pentapeptide isolated from fungus Aspergillus sp. SCSIO 41501

**DOI:** 10.1186/s12985-020-01322-0

**Published:** 2020-04-01

**Authors:** Zhaoyang Wang, Jiaoyan Jia, Lu Wang, Feng Li, Yiliang Wang, Yuzhou Jiang, Xiaowei Song, Shurong Qin, Kai Zheng, Ju Ye, Zhe Ren, Yifei Wang, Shuhua Qi

**Affiliations:** 1 Guangzhou Jinan Biomedicine Research and Development Center, National Engineering Research Center of Genetic Medicine, Jinan University, Guangzhou, Guangdong China; 2 School of Pharmaceutical Sciences, Health Science Center, Shenzhen University, Shenzhen, China; 3 Key Laboratory of Plant Chemistry in Qinghai-Tibet Plateau, Qinghai University for Nationalities, Xining, 810007 Qinghai China; 4 CAS Key Laboratory of Tropical Marine Bio-resources and Ecology, South China Sea Institute of Oceanology Chinese Academy of Sciences, 164 West Xingang Road, Guangzhou, 510301 Guangdong China

**Correction to: Virol J (2020) 17:41**

**https://doi.org/10.1186/s12985-020-01315-z**

Following publication of the original article [1], we have been notified that there is a typo in the title of this article.

Incorrect typo: **pentapepetide**

Correct typo: **pentapeptide**

The original article has been corrected.
